# Expression of pluripotent stem cell markers in mouse uterine tissue during estrous cycle

**Published:** 2016-09-15

**Authors:** Kolsum Choobineh, Saeed Zavareh, Mojdeh Salehnia, Mohamad Taghi Ghorbanian, Seyed Hassan paylakhi

**Affiliations:** 1 *Department of Cellular and Molecular Biology, School of Biology, Damghan University, Damghan, Iran; *; 2 * Institute of Biological Sciences, Damghan University, Damghan, Iran; *; 3 *Department of Anatomy, Faculty of Medical Sciences, Tarbiat Modares University, Tehran, Iran.*

**Keywords:** Estrous Cycle, Mice, Pluripotent Stem Cells, Uterine Tissue

## Abstract

It was assumed that uterine stem cells are responsible for the unique regenerative capacity of uterine. Therefore, the aim of the present study was to investigate the expression of the pluripotent stem cell markers in the mice uterine tissue during different stages of estrous cycles. Twelve virgin female NMRI mice (6 to 8 weeks old) were considered at proestrus, estrus, metestrus and diestrus according to the cell types observed in the vaginal smear and underwent hysterectomy operation. Quantitative real-time polymerase chain reaction (PCR) and immunohistochemical staining for pluripotent stem cell markers (SOX2, OCT4, KLF4, and NANOG) were performed. Immunofluorescence staining revealed that expression and localization of the pluripotency markers SOX2, OCT4, KLF4, and NANOG at the protein level were not different throughout estrous cycle. Also, mRNA of pluripotency markers was detected in all tested samples. However, there were no significant differences in their genes expression at each stage and during the estrous cycle. Different hormonal profile during the estrous cycle could not affect expression of pluripotent stem cell markers in uterine tissue.

## Introduction

Uterine is a unique tissue in its amazing cellular turnover within each reproductive cycle.^1^ In menstruating species, human, the endometrium undergoes cycles of regeneration, differentiation and shedding while, in non-menstruating species, rodents, growth and apoptosis take place during the estrus cycle which is assumed that uterine stem cells provide a source for cellular production in this cyclical regenerating tissue.^1^^-^^4^

Stem cells are rare undifferentiated cells with high proliferative, self-renewal and differentiation potentials. The presence of stem cells has been demonstrated by numerous putative stem cell markers.^1^^,^^5^^-^^7^ Stem cells are classified as totipotent, pluripotent, oligopotent and uni-potent based on their developmental potential.^8^ Pluripotent stem cells are rare and generally small in number and can differentiate into cell line of all three germ layers, however, not the extra-embryonic tissues.^9^^,^^10^ Some investigations have showed evidence for the existence of pluripotent stem cells in uterine tissue.^1^^,^^11^^-^^13^

Many transcription factors are critically involved in the maintenance of stem cells pluripotency. Among them, octamer binding transcription factor 4 (OCT4), SRY-related HMG-box 2 (SOX2), nanog homeobox (NANOG) and Kruppel like factor 4 (KLF4) are thought to be the main regulators.^13^ The OCT4 is a homeodomain transcription factor of the POU family that has been demonstrated to be necessary for regulating self-renewal of undifferentiated embryonic stem cells and preservation of pluripotency. Therefore, it is commonly used as a marker for undifferentiated pluripotent stem cells.^14^ The OCT4 expression must be strongly regulated for preventing differentiation and for sustaining embryonic stem cell self-renewal.^14^ SOX2, as a member of the SOX gene family is necessary for preserving pluripotency of stem cells that works in collaboration with OCT4 to activate transcription of key pluripotency factors.^15^ Moreover, SOX2 in embryonic stem cells is controlling OCT4 expression, therefore, they regulate expression of each other, when expressed concurrently.^16^ NANOG is thought to be a key factor associated with other factors such as OCT4 and SOX2 establishing pluripotency.^17^ The OCT4, SOX2 and NANOG positively regulate transcription of all pluripotency circuitry proteins in the leukemia inhibitory factor (LIF), a key cytokine for maintenance of embryonic stem cell pluripotency, pathway.^15^ The KLF4 has been shown to be highly expressed in undifferentiated embryonic stem cells and its expression decreases significantly during differentiation. Therefore, it has been suggested that Klf4 is very likely an important regulator of stem cell self-renewal and pluripotency.^18^

The mice estrous cycle lasts approximately 4 to 5 days and consists of four stages: Proestrus, estrus, metestrus and diestrus. Since, mice are non-seasonal polyestrous, diestrus is followed by the proestrus phase of the next cycle. The peak of circulating levels of 17b-estradiol (E2) occurs at estrus, whereas progesterone (P4) levels rise during metestrus and diestrus, and then decline from proestrus to estrus.^19^^,^^20^ Sex steroid hormones, E2 and P4, are responsible for a sequential series of cellular proliferation, apoptosis and differentiation, along with extracellular matrix turnover, angiogenesis, and leukocyte infiltration in both humans and mice uterine tissue during reproductive cycles.^21^

To the best of our knowledge, there is no report on expression pattern of pluripotent stem cell during the estrous cycle. The present study was aimed to investigate whether expression of pluripotent stem cell markers (SOX2, OCT4, NANOG and KLF4) can be affected by stages of estrous cycles or not.

## Materials and Methods

**Animals. **Adult virgin female NMRI mice (n = 12; 6 to 8 weeks old) were cared and housed in standard cages in a controlled temperature (22 to 24 ˚C) and humidity (30 to 70%) room, with a 12 hr light: 12 hr dark cycle, lights on at 6:00 a.m. Standard laboratory chow and water were provided *ad libitum*. Animals were allocated to familiarize at least for 10 days before any experimental manipulations were initiated. All the procedures were approved by the Institute of Biological Sciences Ethical Committee for Animal Research, Damghan Univercity, Damghan, Iran.

**Detection of estrous cycle. **Only mice with regular estrous cycles were used for vaginal cytology. The stage of estrous was determined by cytological evaluation of vaginal smears as described previously with some modifications.^22^ Briefly, every morning each animal was examined for vaginal appearance prior to vaginal cytology. Vaginal smears were obtained by a plastic pipette filled with 10 µL of phosphate buffer saline (PBS; Sigma Aldrich, Cambridge, UK) by inserting the tip into the vagina and repeated pipetting and flushing. Vaginal fluid was placed on glass slides. All slides were then fixed and stained with methanol and methylene blue (2%), respectively. Stained slides were observed under a light microscope (Nikon, Tokyo, Japan) with 10 and 40× objective lenses. Estrous cycle stages then were determined using the cellular composition and the relative ratio between the cell types. Three types of cells could be recognized as round and the nucleated cells were epithelial cells, irregular cells without nucleus were the cornified cells and the little round cells were the leukocytes.

**Hysterectomy operation. **Hysterectomy operation was performed by a method described previously.^23^ The anesthetized mice using 10 mg kg^-1^ xylazine (Alfasan, Woerden, The Netherlands) and 100 mg kg^-1 ^ketamine (Rotexmedica, Trittau, Germany) were placed in dorsal recumbency. A mid-ventral incision was made from just cranial to the urethral opening to the abdominal midpoint. The uterine horns were tracked cranially to identify the ovaries. A sterile silk ligature was placed around the ovarian vasculature between the oviduct and uterine horn and the anterior end of each uterine horn was incised from the oviduct. The ovary and oviduct remained in the peritoneal cavity. A sterile silk ligature was placed around the cervix. The cervix was incised cranially to the ligature, and the uterus and both uterine horns were excised. The abdominal muscle wall was closed with catgut suture. Silk sutures were used to close the skin incision.

**Staining of uterine tissue. **After surgery one-third of the uterine horns was collected and fixed with 10% formaldehyde. After that, the samples were washed and dehydrated in an ethanol series of ascending concentration, cleared in xylene (Merck, Darmstadt, Germany), and embedded in paraffin wax (Merck). Then the transverse sections of tissue at 5 µm thickness were stained with hematoxylin and eosin (H & E) to confirm the accuracy of the estrous cycle according to established histological criteria which were described previously.^24^

**Reverse transcription and quantitative real-time PCR. **Total RNA from uterine tissues (three sample per group) was isolated using the RNX^TM^-plus Kit (Cinnagen, Tehran, Iran) according to the manufacturer’s instruction. RNA preparations were DNase I (Cinnagen) treated to eliminate any DNA contamination. RNA quality was assessed using density ratio of 28S to 18S rRNA bands. First strand cDNA synthesis from 1000 ng of total RNA was performed using avian myeloblastosis virus reverse transcriptase (Cinnagen) primed by random hexamers according to manufacturer's instructions. Real-time PCR was performed on a Rotor-Gene 6000 machine (Corbett Life Science, Corbett, USA) using the Quanti Fast SYBR Green PCR Kit (Qiagen, Germantown, USA). DNA was first denatured for 10 min at 94 ˚C, and then amplified using 40 cycles of 15 Sec at 94 ˚C, 25 sec at 60 ˚C, and 30 sec at 72 ˚C. The specificity of PCR products was confirmed by both melting curve analysis and agarose gel electrophoresis. Glyceraldehyde 3 phosphate dehydrogenase (GAPDH) was used as a control gene in the real time PCR experiments. Detailed information on all primers is provided in Table 1. PCR efficiency for each gene was determined according to standard curves. Relative quantification analysis was performed using the 2^-ΔΔCT^ method,^25^ with the Rotor Gene 6000 series software (version 1.7; Corbett Life Science, Corbett, USA). Primer sequences are listed in Table 1.

**Table 1:** Oligonucleotide primer sequences for real time (RT) PCR

**Gene**	**Length (bp)**	**Forward primer**	**Reverse primer**	**Function**
**OCT4**	23	CCTGCAGAAGGAGCTAGAACAGT	TGTTCTTAAGGCTGAGCTGCAA	Marker
**NANOG**	22	TGTGTGCACTCAAGGACAGGTT	TCAGGTTCAGAATGGAGGAGAGTT	Marker
**SOX2**	23	GCACATGAACGGCTGGAGCAACG	TGCTGCGAGTAGGACATGCTGTAGG	Marker
**KLF4**	21	GAAATTCGCCCGCTCCGATGA	CTGTGTGTTTGCGGTAGTGCC	Marker
**GAPDH**	23	TGACATCAAGAAGGTGGTGAAGC	CCCTGTTGCTGTAGCCGTATTC	Housekeeping Gene

**Immunohistochemistry. **Immunohistochemistry was done based on a method described previously with some modifications.^26^ In brief, uterine horn was embedded and frozen in OCT compound (Tissue Tek; Miles Scientific, Naperville, USA). Frozen blocks were sectioned using a Cryostat (Cryocut 1800; Reichert-Jung, Heidelberg, Germany). Consecutive 7 μm cross-sections were mounted on poly-l-lysine coated slides. The slides were fixed with 4% paraformaldehyde for 20 to 30 min. Then the slides were washed three times with PBS for 15 min and transferred into staining glass containing 1% Triton X-100 (Sigma Aldrich, Cambridge, UK) in PBS for 30 min then were washed with PBS several times. The slides were incubated overnight at 4 ˚C with primary antibodies recognizing OCT4 (Abcam, Cambridge, UK), SOX2 (Abcam), NANOG (Abcam) and KLF4 (Abcam) diluted 1:100, 1:1000, 1:200 and 1:50, respectively, according to the manufacturer’s data sheet. The sections were then warmed up for 30 min at room temperature and they were washed with PBS for 10 min and incubated with fluorescin isothiosianid (FITC; Chemicon, Temecula, USA) conjugated rabbit anti-mouse second antibody for 2 hr at 37 ˚C in dark room. After washing with PBS for 5 min, slides were counterstained with 6-diamidino-2-phenylindole (DAPI; Vector Laboratories Inc., Burlingame, USA), to identify nuclei and then mounted with glycerin-PBS (1:1, v/v). In negative controls the primary antibodies were omitted. A fluorescent microscope (Nikon, Tokyo, Japan) was used to evaluate the slides. All the slides were evaluated for the five different tissue compartments, luminal epithelial, glandular epithelia, stroma, myo-metrium and blood vessels. Analyses were performed independently by two blinded investigators.

**Statistical analysis. **All experiments were repeated at least three times. Statistical analysis was performed using SPSS (version 19; SPSS Inc., Chicago, USA), and one way ANOVA for real-time PCR results. Post hoc Tukey's HSD was used for multiple comparisons. A *p*-value less than 0.05 was considered statistically significant.

## Results

**Fig. 1:**
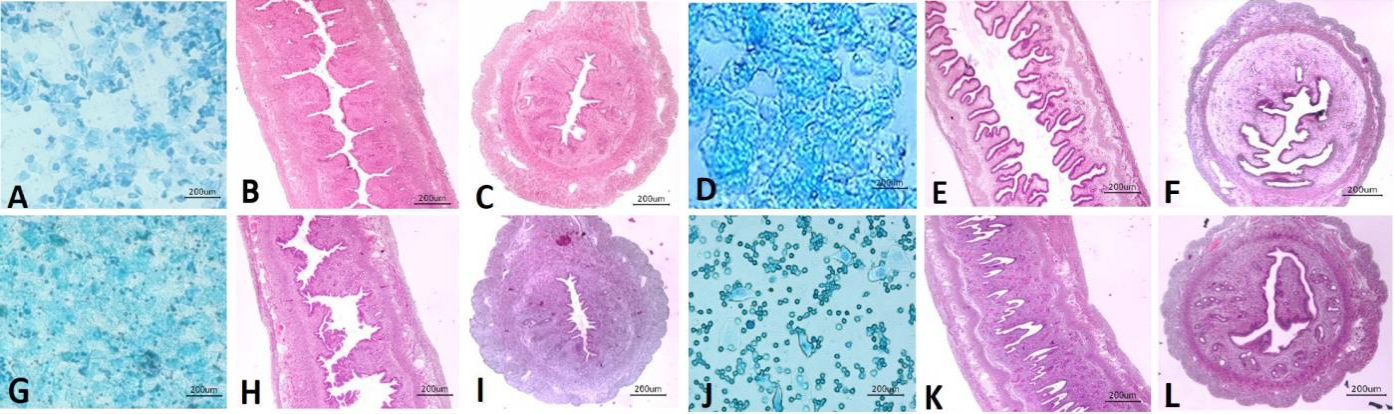
The stained vaginal smears with methylene blue and longitudinal and cross section of uterus (H & E). A; Proestrous smear consists of a majority of nucleated epithelial cells. B and C: Proestrous; Endometrial epithelial cells are cuboidal to columnar, slight leukocyte infiltration and luminal dilatation can be seen. D: Estrous smear mainly consists of anucleated cornified cells. E and F: Estrous: Endometrial epithelial cells are large and tall columnar. Mitotic figures are rare and lumen dilatation can be detected with high numbers of leukocyte infiltrated in the lamina propria and endometrial glands. G: Metestrous smear consists of the equal proportion among leukocytes, cornified and nucleated epithelial cells. H and I: Metestrous: reduced height of endometrial epithelium of metestrous uterine, increased mitotic activity and leukocyte infiltration can be seen. J: Diestrous smear consists a high proportion of inflammatory cells; leukocytes. K and L; Diestrous: Uterine lumen slit-like and lined with small endometrial epithelial cells, numerous mitotic figures and stromal edema were present

The cytological appearance of cells recovered from mouse vaginal washing and uterine histological features in order to determine the four stages of estrous cycle were shown in Figure 1. At preostrous stage, the vaginal opening was swollen, wet and reddish pink. In addition, vaginal smear obtained during proestrous phase consisted of primarily nucleated epithelial cells, few cornified epithelial cells and few leukocytes (Fig. 1A). Histological features of the proestrous uterine tissue showed that, the endometrial epithelium cells were cuboidal to columnar. Mitoses signs were present in luminal uterine epithelium with little or without degeneration. Also, there were slight leukocyte infiltration and luminal dilatation (Figs. 1B and 1C). At the estrous stage, vaginal opening turned to be lighter pink, less humid, and less swollen and the cytological appearance of vaginal smear showed primarily anucleated cornified epithelial cells (Fig. 1D). Histological appearance of estrous uterine demonstrated that endometrial layer composed of large and tall columnar cells. Noticeable degeneration of epithelial cells was detected. Mitotic figures were rare and lumen dilatation was present. Also, high numbers of leukocyte were infiltrated in the lamina propria and endometrial glands (Figs. 1E and 1F). At the metestrus stage the vaginal opening was not open and wide. Also, the vaginal appearance was pale and not swollen. An equal proportion among leukocytes, cornified, and nucleated epithelial cells were detected in vaginal smears of metestrus mouse (Fig. 1G). In comparison with estrous stage the height of endometrial epithelium of metestrus uterine was reduced, mitotic activity increased and leukocyte infiltration was rarely seen. Also degeneration of endometrial epithelial cells was persisted (Figs. 1H and 1I). At the diestrus stage, the vaginal opening was small and closed with no swelling. Furthermore, high proportion of leukocytes, some nucleated epithelial cells, and mucus were seen in vaginal smears of diestrus stage (Fig. 1J). The uterine lumen of mice at diestrus stage was small, avascular and slit-like which was lined with small endometrial epithelial cells. Also numerous mitotic figures were detected and stromal edema was present. Also, there were sporadic degenerated cells, and low numbers of leukocytes within the lamina propria (Figs. 1K and 1L).

**Fig. 2:**
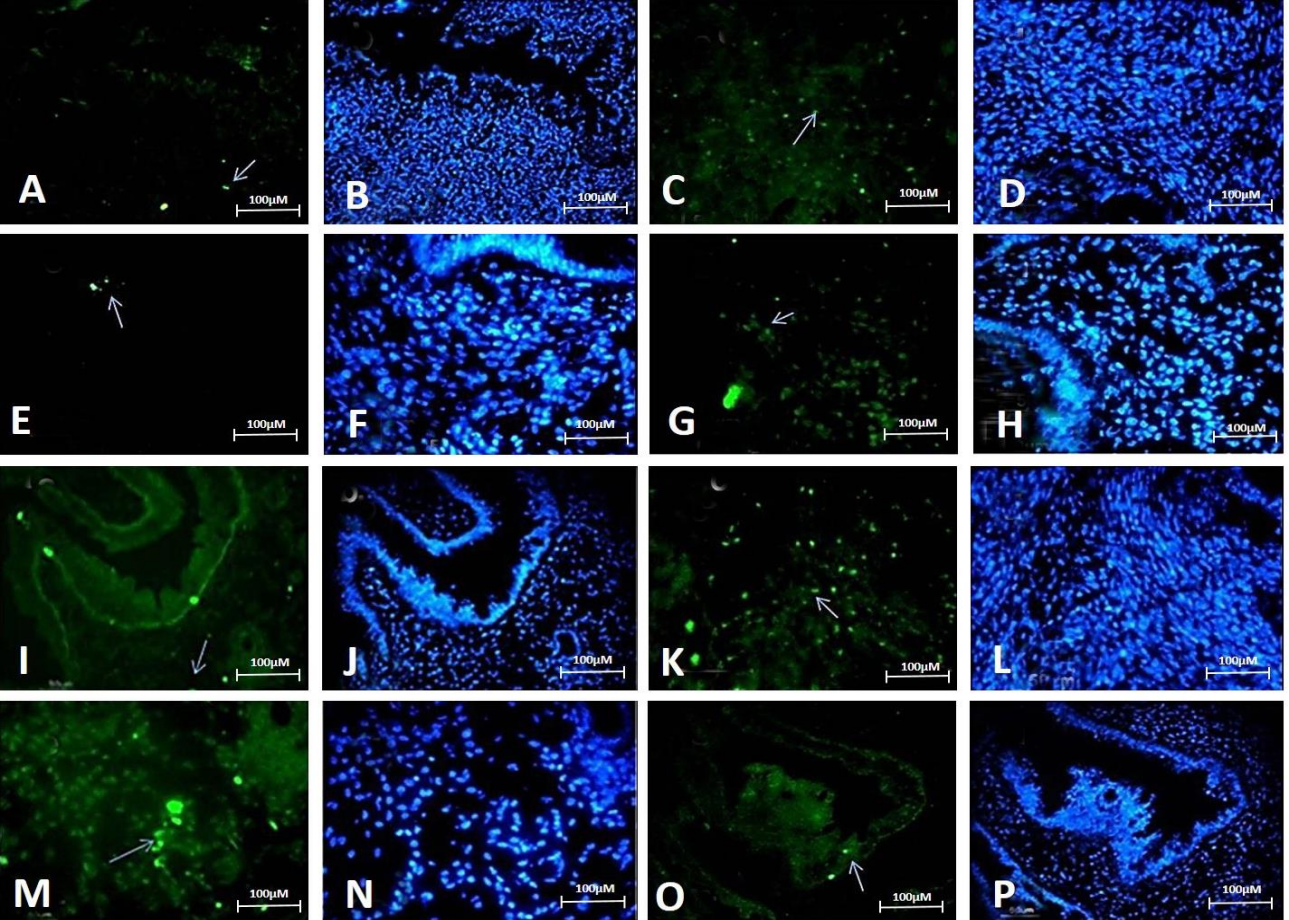
Expression of pluripotent stem cell markers (SOX2, OCT4, NANOG and KLF4) in uterine tissue at proestrous and estrous stages. White arrow indicate nucleus of positive cells. A and B: KLF4 at proestrous stage, C and D: NANOG at proestrous stage, E and F: OCT4 at proestrous stage, G and H: SOX2 at proestrous stage, I and J: KLF4 at estrous stage, K and L: NANOG at estrous stage, M and N: OCT4 at estrous stage, O and P: SOX2 at estrous stage

**Fig. 3:**
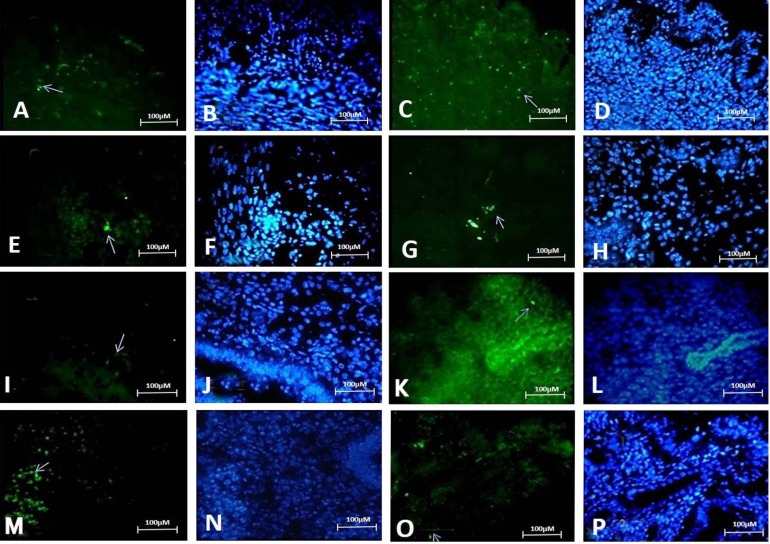
Expression of pluripotent stem cell markers (SOX2, OCT4, NANOG and KLF4) in uterine tissue at metstrous and distrous stages. White arrow indicate nucleus of positive cells. A and B: KLF4 at metestrous stage, C and D: NANOG at metestrous stage, E and F: OCT4 at metestrous stage, G and H: SOX2 at metestrous stage, I and J: KLF4 at diestrous stage, K and L: NANOG at diestrous stage, M and N: OCT4 at diestrous stage, O and P: SOX2 at diestrous stage

Immunohistochemistry study demonstrated expression of the pluripotency markers SOX2, OCT4, KLF4, and NANOG in different consecutive cross-sections of the mouse uterine tissue. Positive cells were frequently found in endometrial glands, endometrial stroma, and perivascular areas of the basal and functional layers of endometrium at proestrus, estrous, metestrus and diestrus stages (Figs. 2 and 3).

**Fig. 4:**
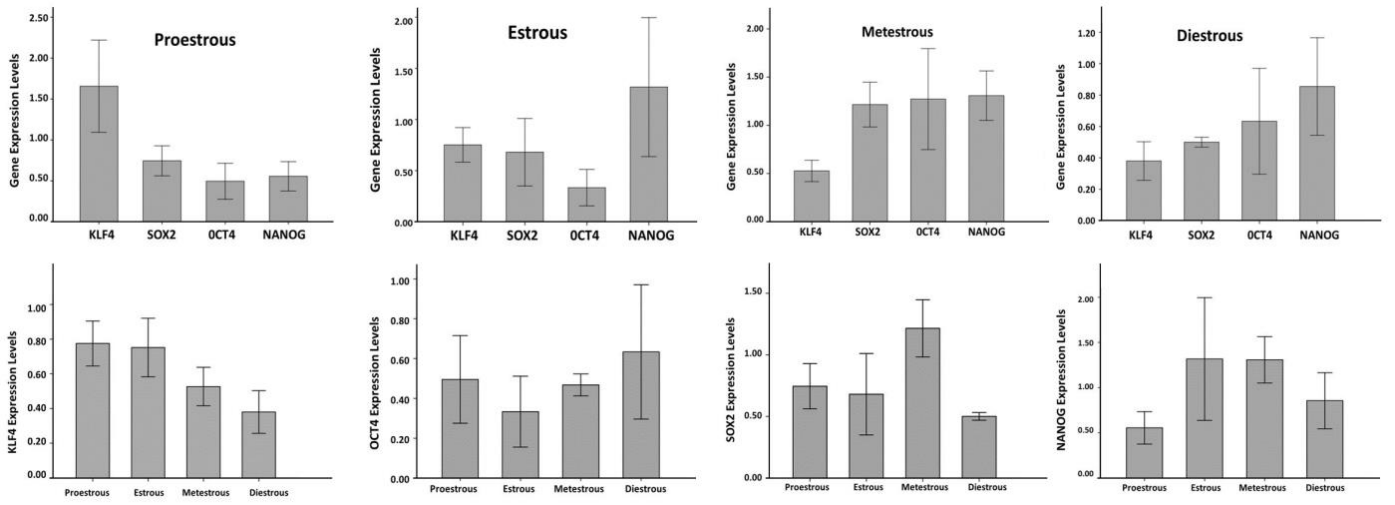
Real Time RT-PCR results of expression KLF4, SOX2, OCT4 and NANOG at different stage of estrous cycle. Comparison of the gene expression levels revealed no significant differences among SOX2, NANOG, KLF4, and OCT4 at different stages of estrous cycles (*p* > 0.05

Expression of pluripotency markers SOX2, NANOG, KLF4, and OCT4, was analyzed by real-time (RT) PCR and calculated by the 2^-ΔΔct^ formula. As shown in Figure 4, mRNA of pluripotency markers was detectable in all stages of estrous cycle. Although the mRNA quantities were varied, comparison of the gene expression levels revealed no significant differences among SOX2, NANOG, KLF4, and OCT4 at different stages of estrous cycles (*p* > 0.05).

## Discussion

Circulating levels of sex steroid hormones during the menstrual cycle result in growth, differentiation and shedding of uterine endometrial layer,^27^ whereas, there is little change in the basal layer of uterine tissue and relatively insensitive to changes in hormone levels.^28^ It seems that the special cell population in this area is responsible for endometrial regeneration. As soon as the hormonal levels decrease, the endometrium does not support, and menstruation occurs as a result of necrosis of the endometrium.^11^ The uterine endometrium undergoes cycles of growth and apoptosis instead of bleeding in the species that do not have menstrual cycle.^2^

Growth of new tissue is similar to the cellular turnover in the bone marrow that adult stem cells are responsible for cell production and reconstruction. Therefore, it was assumed that adult stem cells may have key role in reconstructing the functional layer of endometrium.^2^^,^^11^ Several studies have shown the stem cells in the uterus, while, there was not any report about an expression pattern of stem cell markers during estrous cycles. Hence, the aim of the present study was to investigate whether the expression of pluripotent stem cell markers, SOX2, OCT4, NANOG and KLF4, can be affected by stages of estrous cycles or not.

Immunohistochemistry is a precise method to determine the position of the tissue-specific antigens. Thus, in this study, the position of pluripotent stem cell markers of the mouse uterus was investigated using this technique. Results showed that SOX2, OCT4, NANOG and KLF4 were expressed in the endometrium and myometrium of the mouse uterus at the all phases of the estrous cycle.

Positive cells for OCT4 were found in the stroma of all the samples which were frequently located in peri-glandular position. These results were in agreement with mattahi *et al*. who demonstrated the presence of OCT4 positive cells in human endometrial tissue.^29^ The amount of intracellular OCT4 controls the fate of differentiation, mainly in embryo stem (ES) cells.^14^ The quantity of OCT4 was analyzed in the different stage of estrous cycles by real time PCR. For a long time, OCT4 was accepted as a reliable marker to show pluripotent stem cells but it was known that OCT4 alone do not enough because there were various isoform of OCT4.^30^ Thus, in this study other markers such as SOX2, NANOG and KLF4 were used.

Our results showed that SOX2 positive cells were frequently present in perivascular location which was in agreement with results of Gotte *et al*. SOX2 is a critical factor for keeping the stemness of neural stem cells and then of ES cells.^13^ SOX2 in combination with OCT4 and NANOG operate as a main regulator of mammalian embryogenesis. Also, it was shown that SOX2 is a portion of a complex network of transcription factors that affects both pluripotency and differentiation in ES cells.^31^

In addition, our study demonstrated expression of KLF4 and NANOG in mouse uterine tissue during the estrous cycle. The KLF4 is a member of the KLF family of transcription factors and regulates proliferation, differentiation, apoptosis and somatic cell reprogramming. Evidence also suggests that KLF4 is a tumor suppressor in certain cancers.^32^ Also, NANOG has been suggested to be one of four major factors that control reprogramming of adult cells into germ-line-competent induced pluripotent stem cells.^33^ In agreement, Gotte *et al*. showed expression of the pluripotency factors SOX2, OCT4, KLF4, and NANOG y in the human endometrium, myometrium, and endo-metriotic tissue and suggested that expression of SOX2, OCT4, KLF4, and NANOG marks the endometrium as an attractive potential source for the generation of iPS cells.^13^ Evidences of the presence of adult stem cells in the endometrium has been previously reported.^1^^,^^2^^,^^4^

Whereas, origin of endometrial stem cells remains blurred. It seems that endometrial stem cells may be derived from embryonic stem cells which remains in adult uterine endometrium tissue and participate in the its reconstruction during menstruation or estrous cycle.^1^^,^^2^ Based on the presence of a small number of circulating stem cells of the bone marrow in various organs, it appears that bone marrow is another source of endometrial stem cells.^2^^,^^34^

The results of the present study showed that expression pattern of pluripotent stem cell markers, SOX2, OCT4, NANOG and KLF4 in uterine tissue was similar in the proestrus, estrous, metestrus and diestrus stages with no significant differences. This findings of the present study showed that expression of pluripotent stem cell markers was different within each estrous stage that supports the results of Gotte *et al*. that indicated there was a sex steroid-independent pool of stem cells.^13 ^As immunofluorescent does not apply to determine the exact number of genes expression in the tissue, therefore, real time PCR was used in the next part of this study.

Comparison of KLF4, SOX2, OCT4 and NANOG genes expression in different phases of the estrous cycle, proestrus, estrus, metestrus and diestrus, showed no statistically significant difference. This was in agreement with results of Bentz *et al.* that reported OCT4 expression independent of hormonal changes in the endometrium.^35^ Since, Oct4 is the main regulator of differentiation of pluripotent stem cell line,^10^ this seems to be related to the specific function of this gene. Expression of OCT4 gene is said to be strictly regulated, because excessive or low expression of OCT 4 results in the differentiation of stem cells.^14^ Also, comparison of the expression of pluripotent markers within groups revealed no significant differences among the expression of SOX2, OCT4, NANOG and KLF4 marker genes in proestrus, estrous, metestrus and diestrus separately. It seems that this was also related to the specific function of these genes. It has been showed that OCT4, NANOG and SOX2 working together closely in preventing the expression of developmental genes which causes the cells to remain undifferentiated state.^17^ For example, KLF4 is a factor that inhibits the expression of P53, an inhibitor of NANOG expression. Hence, KLF4 induces expression of the NANOG indirectly.^32^^,^^36^

In conclusion, our findings indicated that normal hormonal changes during the estrous cycle did not affect expression levels of pluripotent stem cell markers of mouse uterine tissue. Further studies are required to clarify the effect of different hormonal profile on the expression of other uterine stem cells markers.
